# Supplementary material for: A modular CRISPR screen identifies individual and combination pathways contributing to HIV-1 latency
Source: PLoS Pathog. 2023 Jan 27;19(1):e1011101. doi: 10.1371/journal.ppat.1011101 (PMC9907829; doi:10.1371/journal.ppat.1011101)
Supplement: S4 Table — (DOCX) [file ppat.1011101.s004.docx]

**Supplemental Table 4**

| **Gene Name** | **Previously implicated in HIV-1 latency?** | **Reference** |
| --- | --- | --- |
| CUL3 | No |  |
| DNMT1 | Yes, conflicting results | [1], [2], [3], [4] |
| ACTL6A | Yes | [5] |
| TAF5 | No |  |
| KAT5 | Yes | [6] |
| YEATS4 | No |  |
| SRCAP | No |  |
| DMAP1 | Yes, conflicting results | [1], [2], [3], [4] |
| VPS72 | No |  |
| MBD2 | Yes | [3] |
| AURKB | Yes | [7] |
| EXOSC9 | No |  |
| CTCF | Yes | [8] |
| NSL1 | No |  |
| SUZ12 | Yes | [9], [10] |
| TAF12 | No |  |
| CDYL | No |  |
| ZNHIT1 | No |  |
| MSL2 | No |  |
| RNF20 | No |  |
| DPY30 | No |  |
| INTS12 | No |  |
| WR5 | No |  |
| EXOSC2 | No |  |
| KAT8 | Yes, conflicting results | [6], [11] |
| EXOSC4 | No |  |
| KMT2D | No |  |
| ING3 | No |  |
| MCM2 | No |  |
| ARID1A | Yes | [12] |

**Supplemental References**

1. Blazkova J, Trejbalova K, Gondois-Rey F, Halfon P, Philibert P, Guiguen A, et al. CpG methylation controls reactivation of HIV from latency. PLoS Pathog. 2009;5(8):e1000554. Epub 2009/08/22. doi: 10.1371/journal.ppat.1000554. PubMed PMID: 19696893; PubMed Central PMCID: PMCPMC2722084.

2. Trejbalová K, Kovářová D, Blažková J, Machala L, Jilich D, Weber J, et al. Development of 5' LTR DNA methylation of latent HIV-1 provirus in cell line models and in long-term-infected individuals. Clin Epigenetics. 2016;8:19. Epub 2016/02/24. doi: 10.1186/s13148-016-0185-6. PubMed PMID: 26900410; PubMed Central PMCID: PMCPMC4759744.

3. Kauder SE, Bosque A, Lindqvist A, Planelles V, Verdin E. Epigenetic regulation of HIV-1 latency by cytosine methylation. PLoS Pathog. 2009;5(6):e1000495. Epub 2009/06/27. doi: 10.1371/journal.ppat.1000495. PubMed PMID: 19557157; PubMed Central PMCID: PMCPMC2695767.

4. Blazkova J, Murray D, Justement JS, Funk EK, Nelson AK, Moir S, et al. Paucity of HIV DNA methylation in latently infected, resting CD4+ T cells from infected individuals receiving antiretroviral therapy. J Virol. 2012;86(9):5390-2. Epub 2012/02/22. doi: 10.1128/jvi.00040-12. PubMed PMID: 22345448; PubMed Central PMCID: PMCPMC3347337.

5. Van Duyne R, Guendel I, Narayanan A, Gregg E, Shafagati N, Tyagi M, et al. Varying modulation of HIV-1 LTR activity by Baf complexes. J Mol Biol. 2011;411(3):581-96. Epub 2011/06/28. doi: 10.1016/j.jmb.2011.06.001. PubMed PMID: 21699904; PubMed Central PMCID: PMCPMC3146592.

6. Li Z, Mbonye U, Feng Z, Wang X, Gao X, Karn J, et al. The KAT5-Acetyl-Histone4-Brd4 axis silences HIV-1 transcription and promotes viral latency. PLoS Pathog. 2018;14(4):e1007012. Epub 2018/04/24. doi: 10.1371/journal.ppat.1007012. PubMed PMID: 29684085; PubMed Central PMCID: PMCPMC5933813.

7. Nunes JM, Furtado MN, de Morais Nunes ER, Sucupira MCA, Diaz RS, Janini LMR. Modulation of epigenetic factors during the early stages of HIV-1 infection in CD4(+) T cells in vitro. Virology. 2018;523:41-51. Epub 2018/08/06. doi: 10.1016/j.virol.2018.07.026. PubMed PMID: 30077875.

8. Jefferys SR, Burgos SD, Peterson JJ, Selitsky SR, Turner AW, James LI, et al. Epigenomic characterization of latent HIV infection identifies latency regulating transcription factors. PLoS Pathog. 2021;17(2):e1009346. Epub 2021/02/27. doi: 10.1371/journal.ppat.1009346. PubMed PMID: 33635929; PubMed Central PMCID: PMCPMC7946360.

9. Nguyen K, Das B, Dobrowolski C, Karn J. Multiple Histone Lysine Methyltransferases Are Required for the Establishment and Maintenance of HIV-1 Latency. mBio. 2017;8(1). Epub 2017/03/02. doi: 10.1128/mBio.00133-17. PubMed PMID: 28246360; PubMed Central PMCID: PMCPMC5347344.

10. Potjewyd F, Turner AW, Beri J, Rectenwald JM, Norris-Drouin JL, Cholensky SH, et al. Degradation of Polycomb Repressive Complex 2 with an EED-Targeted Bivalent Chemical Degrader. Cell Chem Biol. 2020;27(1):47-56.e15. Epub 2019/12/14. doi: 10.1016/j.chembiol.2019.11.006. PubMed PMID: 31831267; PubMed Central PMCID: PMCPMC7004250.

11. Huang H, Kong W, Jean M, Fiches G, Zhou D, Hayashi T, et al. A CRISPR/Cas9 screen identifies the histone demethylase MINA53 as a novel HIV-1 latency-promoting gene (LPG). Nucleic Acids Res. 2019;47(14):7333-47. Epub 2019/06/06. doi: 10.1093/nar/gkz493. PubMed PMID: 31165872; PubMed Central PMCID: PMCPMC6698651.

12. Marian CA, Stoszko M, Wang L, Leighty MW, de Crignis E, Maschinot CA, et al. Small Molecule Targeting of Specific BAF (mSWI/SNF) Complexes for HIV Latency Reversal. Cell Chem Biol. 2018;25(12):1443-55.e14. Epub 2018/09/11. doi: 10.1016/j.chembiol.2018.08.004. PubMed PMID: 30197195; PubMed Central PMCID: PMCPMC6404985.
