# Supplementary figures and images for: A modular CRISPR screen identifies individual and combination pathways contributing to HIV-1 latency
Source: PLoS Pathog. 2023 Jan 27;19(1):e1011101. doi: 10.1371/journal.ppat.1011101 (PMC9907829; doi:10.1371/journal.ppat.1011101)

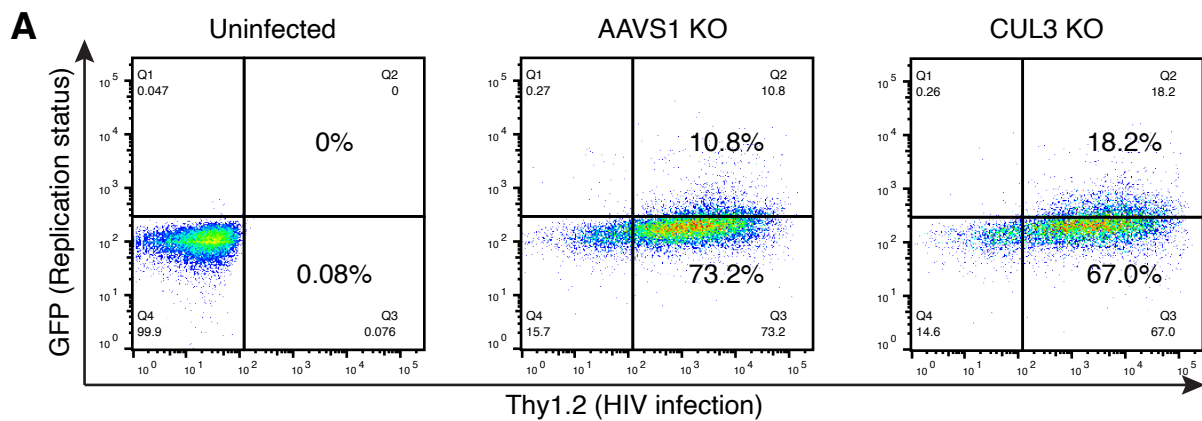

Supplement: S1 Fig — (A) Representative flow cytometry plots of viral reactivation levels in wildtype primary CD4+ T cells and primary CD4+ T cell model of HIV-1 latency cells upon knockout of AAVS1 and CUL3. Thy1.2-, GFP- cells (quadrant 4) are uninfected; Thy1.2+, GFP- (quadrant 3) cells are infected with the dual reporter HIV-1 virus and latent; Thy1.2+, GFP+ cells (quadrant 2) are infected and reactivated. (PDF) [file ppat.1011101.s009.pdf]

**A**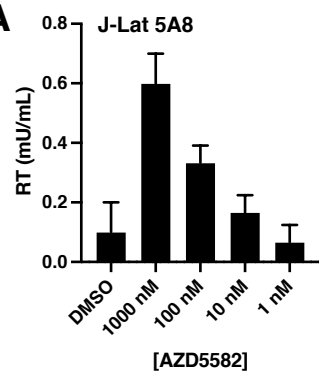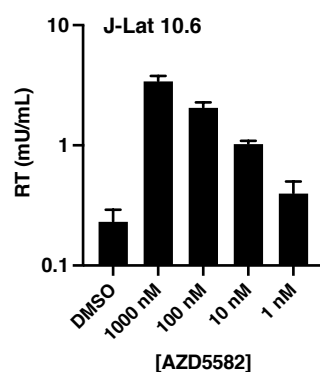

Supplement: S2 Fig — (A) AZD5582 dose curve performed on both J-Lat 10.6 and 5A8 cell lines to determine viral reactivation levels. (PDF) [file ppat.1011101.s010.pdf]

A

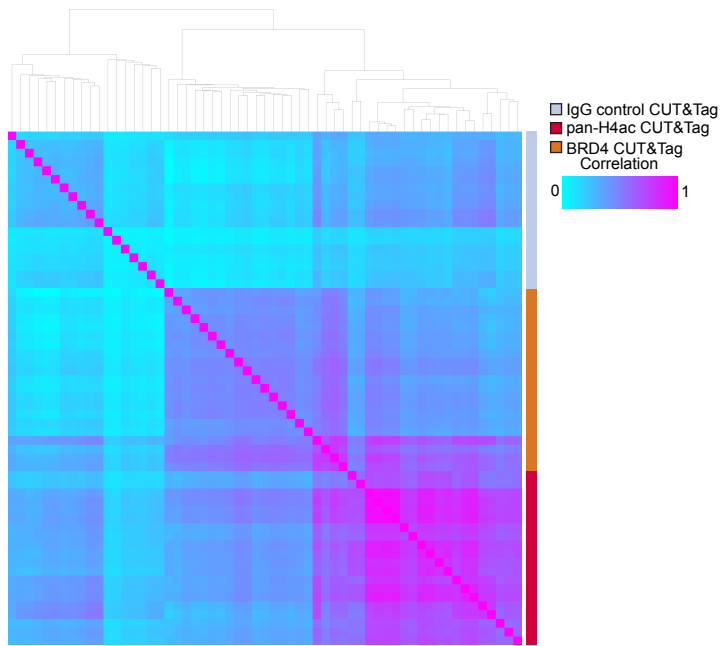

B

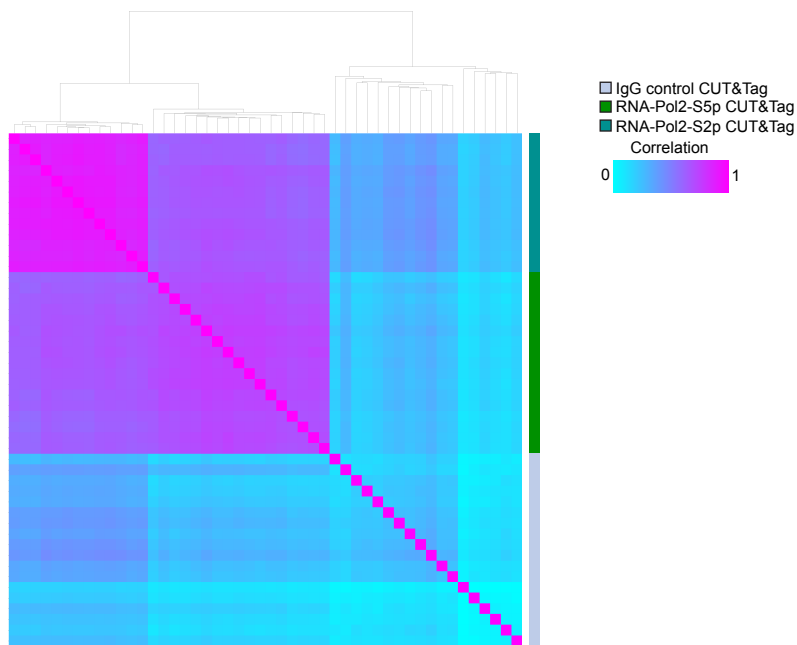

Supplement: S3 Fig — (A) Correlation Matrix colored according to the pair-wise Pearson correlation of pan-H4Ac, BRD4, and IgG negative control samples across the merged pan-H4Ac and BRD4 peak sets. All pan-H4Ac samples group together by hierarchical clustering as do all of the BRD4 samples. (B) Same as (A) but showing the pair-wise Pearson correlation of RNA-Pol2-S5p and RNA-Pol2-S2p over the merged peak sets of these marks. (PDF) [file ppat.1011101.s011.pdf]
