# Supplementary material for: A modular CRISPR screen identifies individual and combination pathways contributing to HIV-1 latency
Source: PLoS Pathog. 2023 Jan 27;19(1):e1011101. doi: 10.1371/journal.ppat.1011101 (PMC9907829; doi:10.1371/journal.ppat.1011101)
Supplement: Attachment — Submitted filename: RebuttalLetter_v4.pdf [file ppat.1011101.s012.pdf]

Reviewer’s Comments

Reviewer #1: 1. The description of the screen design and conduct in the Results section does not specify that both J-Lat models used were actually clonal ZAP knock-outs. To the general reader this might be somewhat misleading, especially since later CRISPR knock-outs are specified to be pools of cells to “avoid clone-to-clone differences” (Lines 215-216). Although the generation of ZAP knock-out cells is described in the Materials and Methods, this is an important piece of information regarding the screen and the fact that modified J-Lats were used for these screens should be stated in the main text.

We have now moved up the description of the *ZAP* knockouts to the main text (Line 164-168). The starting cells for the screens are indeed clonal lines in which *ZAP* has been knocked out. The use for pools of knockout cells instead of clones rather refers to the validation experiments. We agree that this was awkwardly described in the original and have taken out the phrase “avoid clone-to-clone differences” and instead refer directly to the data in Figure 4A for the degree of knock-out of each pool.

2. A clearer description of what has already been determined regarding the factors identified here would be helpful to put this investigation in context with the existing literature. For readers that are not experts in HIV-1 latency, it may be somewhat confusing what genes listed in Figure 3 have previously been identified versus those that were unique to this screen.
Perhaps a supplemental table with references for the genes shown in Figure 3B would be appropriate.
Did the authors find any novel factors in the screens without LRA addition?
Are all the factors shown in Figure 4 previously implicated in HIV-1 latency?

This is a good suggestion. We have now added a table (Supplemental Table 4) that describes which of the genes in Figure 3 were previously implicated in HIV latency (along with a reference), and which are novel hits. The majority of the factors we identify without LRA addition are indeed novel factors. We specifically chose the novel factors, along with a few positive controls, to validate in Figure 4. We now more clearly describe which factors are novel and which were previously identified and included as a positive control in Figure 4. In addition, we have now added a paragraph in the Discussion (Line 579-609) to expand on some of the top hits in the screen of Figure 3 that were validated in Figure 4.

3. The results concerning the role of ING3 in primary cells presented in Figure 6B-C do not coincide with the results from J-Lats, but the discrepancy is not discussed in the text. Specifically, there is no significant difference between DMSO and AZD treatment in the ING3 knock-out cells, as opposed to the large differences shown in Figure 6A. While the results in primary cells are not necessarily expected to fully recapitulate the J-Lat model, the authors should at a minimum note the discrepancy and ideally discuss potential explanations.

We agree that the differences between primary cells and J-Lat cells were not adequately discussed. We now point out that primary cells are less sensitive to activation by AZD5582 alone than J-Lat cells. However, the important point is that the combination of AZD5582 PLUS knockout of *ING3* activates proviruses in primary cells. We have now added a paragraph in the Discussion (in Lines 610-631) to discuss the differences more fully in magnitude of activation between the primary cells and the J-Lat models

Since the paper was originally submitted, we have also repeated the primary cell experiment with one additional donor. This additional data has been added to Figure 6C. It does not change the result, but adds to the statistical significance of the differences between DMSO control and AZD5582 plus *ING3* knockout in the primary cells.

Reviewer #2: The relevance of the manuscript greatly depends how good the Jurkat cell model reflects latency reversal in primary cells. Therefore, the degree of latency reversal in primary CD4 T cells, as assessed in Fig. 6B, C should be reported and discussed more clearly.

We agree that the differences between primary cells and J-Lat cells were not adequately discussed. We now point out that primary cells are less sensitive to activation by AZD5582 alone than J-Lat cells. However, the important point is that the combination of AZD5582 PLUS knockout of *ING3* activates proviruses in primary cells. We have now added a paragraph in the Discussion (in Lines 610-631) to discuss the differences more fully in magnitude of activation between the primary cells and the J-Lat models

Since the paper was originally submitted, we have also repeated the primary cell experiment with one additional donor. This additional data has been added to Figure 6C. It does not change the result, but adds to the statistical significance of the differences between DMSO control and AZD5582 plus *ING3* knockout in the primary cells.

The representative flow cytometry plots should also contain a graph demonstrating the percentage of Thy1.2+ GFP+ cells before the cells have reached latency (e.g. data after the selection of Thy1.2 positive cells).

Unfortunately, while this would have been a good idea, we did not have enough cells after transduction and bead selection to immediately assess them before reaching latency. The one piece of data we have that partially addresses this is an experiment where we looked at a control knockout line (*AASV1* KO) at both day 13 (day 10 post-selection) before they have reached full latency and at day 19 (day 16 post-selection). As can be seen on the plot below for the reviewer, there is indeed a lower percentage of Thy1.2+, GFP+ cells (Q2, quadrant 2) at day 19 than at day 13, suggesting that as cells enter a resting state, there are more latent proviruses. As this is not a definitive experiment, we would prefer not to include this data in the actual manuscript

It is also unclear, how precisely the „reactivation fold change“ in Fig. 6C was calculated. Is this based on percent GFP+ cells or does it include fluorescence intensities of the GFP+ cell population?

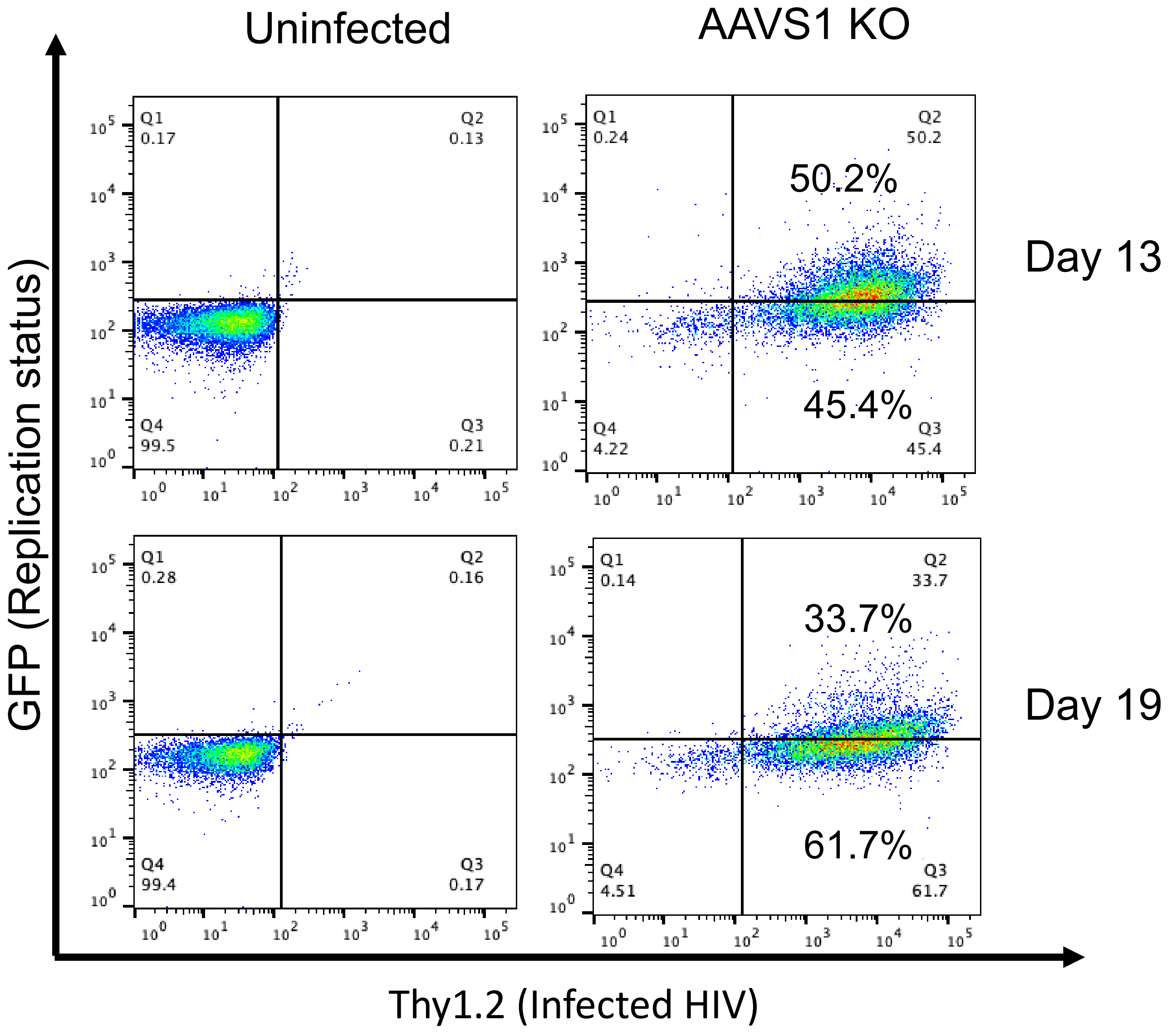

We have now clarified how “reactivation fold change” is calculated in the figure legend of Figure 6C (Line 751-755). The reactivation fold is change is calculated using the percent Thy1.2+, GFP+ cells. The quantified percent Thy1.2+, GFP+ cells for each condition (knockout of *AAVS1* or *ING3* and treatment of DMSO or 1 μM AZD5582) was then normalized to the *AAVS1* knockout with DMSO (negative control) condition. At the reviewer’s suggestion, we also analyzed the data by mean fluorescence intensities of the Thy1.2+, GFP+ cell population, and saw very similar results (we did not add this data to the manuscript).

It should be discussed why the fold-change reported in this assay, is not consistent with „dramatic increase“ stated in line 443 for the CUT + Tag data.

We apologize for the confusion. The fold-change refers to the J-Lat 10.6 cell line that shows an 11-fold increase of virus reactivation with the combination of AZD5582 and *ING3* knockout (as measured by reverse transcriptase activity in the supernatant). The CUT&Tag data is also performed with the J-Lat 10.6 cells. We have clarified the language to make it clear what comparisons we are making.

Reviewer #1: 1. The justification and explanation for what genes were labeled in Figure 3A is excellent, but Figure 5A, which presents similar data, does not include this type of labeling. Although it is clear that the purpose of this figure is to identify factors specific to the screen conducted in the presence of the LRA, it would still be valuable to label genes that meet the same criteria as in 3A.

We have now modified Figure 5A such that all gene names with a cut-off of false discovery rate (FDR) <10% have been labelled. This corresponds to a -log(MaGeCK score) of slightly above 5.

2. Lines 163-164 – How do the authors define what a “significant enrichment” is in reference to Figure 2A? Is there a fold-change cut-off? Is there a way to indicate this in the figure?

Significant enrichment is based on t-test that describes the presence of outliers rather than a fold-change cut-off. We indicate this in the main text and in the figure legend as there is not a way to directly indicate this on the figure.

3.An interaction between CUL3 and NF-κB/NFAT transcription factor binding sites in the HIV-1 LTR has been previously shown (PMID:32882949), it would be worthwhile to briefly discuss how the findings presented here regarding CUL3 relate to this previous report for the overall understanding of how non-canonical NF-κB signaling affects HIV-1 transcription.

Yes, we agree. We now cite this paper in the Discussion in the new paragraph about hits seen in the absence of LRA (Line 579-609).

4.There are several portions of the text that are somewhat difficult to read due to awkward use of past/present tense. For example, Line 262 should read “AAVS1 gene was targeted”. A revision with careful attention to verb tenses would improve readability.

We have done additional copy-editing to correct tense changes.

5.Line 175 – This is strange phrasing, control guides are non-targeting, not “targeting NTCs”.

We have re-phrased this as “compared to the non-targeting sgRNAs.”

6.Line 462 – Is it appropriate to say these factors were “identified” here? Perhaps “verified” or “validated” would be more accurate.

We have changed the language to validated.

7.What is the ICE score for the ING3 knock-out cells shown in Figure 6A?

We have added in ICE KO scores for the pooled *ING3* knockout cells in Figure 6A in the figure legend.

8.There appears to be no p100 band in the ING3 KO 5A8 cells in the absence of AZD treatment as opposed to the control cells. Is there reduced NFKB2 expression in these cells?

Yes, we agree that the p100 band is very faint. We do not have an explanation for this, but now specifically point it out in the text.

9.It is somewhat difficult to identify the colored dots in Figure 8. The authors might consider use of color-blind friendly schemes (such as the Paul Tol schemes) for figures that require the reader to discriminate between data points based on color to interpret points not specifically highlighted by the authors.

Thank you for the suggestion. We have now changed the colors in Figure 8 to a color-blind friendly Paul Tol scheme.

Reviewer #2:
The introduction of latency should also include a reference to silencing mechanisms prior to integration.

We have now added a reference for mechanisms involved in the silencing of unintegrated DNA

Fig. 2B. Legend should state that data points represent individual guide RNAs targeting the same gene

We have updated the figure legend to make this point clear.
Fig. 4 and 6A. The results of the RT-assay may depend on replication of the reactivated provirus. Therefore, it is essential to describe how many days after knock-out RT activity was detemined.

The provirus in the J-Lat cells has a deletion in env so that virions are produced, but they are not infectious and do not spread in the culture. We now point this out on line 238-240. In any case, we also include information on how many days after knock-out the RT assays are done.

Fig. 4 and 6A. The large differences in the degree of RT activation between the two cell clones also deserves a comment

We now comment on this on line 241-244.
